# Quantitative secretomic analysis of pancreatic cancer cells in serum-containing conditioned medium

**DOI:** 10.1038/srep37606

**Published:** 2016-11-21

**Authors:** Peng Liu, Yejing Weng, Zhigang Sui, Yunhao Wu, Xiangli Meng, Mengwei Wu, Haoyi Jin, Xiaodong Tan, Lihua Zhang, Yukui Zhang

**Affiliations:** 1 1st Department of general surgery, Shengjing Hospital, China Medical University, Shenyang 110004, China; 2 Key Lab of Separation Sciences for Analytical Chemistry, National Chromatographic R. & A. Center, Dalian Institute of Chemical Physics, Chinese Academy of Sciences, Dalian 116023, China

## Abstract

Pancreatic cancer is a highly metastatic and chemo-resistant disease. Secreted proteins involved in cell-cell interactions play an important role in changing the tumor microenvironment. Previous studies generally focus on the secretome of cancer cell line from serum-free media, due to the serious interference of fetal bovine serum (FBS). However, serum-starvation may alter expression patterns of secreted proteins. Hence, efforts to decrease the interference of serum in proteomic analysis of serum-containing media have been hampered to quantitatively measure the tumor secretion levels. Recently, the metabolic labeling, protein equalization, protein fractionation and filter-aided sample preparation (FASP) strategy (MLEFF) has been successfully used to avoid the disturbance of serum on secretome analysis. Here, this efficient method was applied for comparative secretome analysis of two hamster pancreatic cancer cells with differentially metastatic potentials, enabling the observation of 161 differentially expressed proteins, including 106 proteins that had been previously reported and detected in plasma. By integrated analysis of our data and publicly available bioinformatics resources, we found that a combination panel consisting of CDH3, PLAU, and LFNG might improve the prognosis of overall pancreatic cancer survival. These secreted proteins may serve as a potential therapeutic targets for pancreatic cancer metastasis.

Pancreatic cancer is the leading cause of cancer-related deaths. Individuals who have pancreatic cancer have a <5% 5-year survival rate. During the past five years, approximately 732,000 Chinese have been diagnosed with pancreatic cancer^1^. Unfortunately, the majority of diagnosed patients are in advanced stages of disease, with local invasion and distant metastasis, and surgery is the only treatment option for them^2^. Therefore, early diagnosis and immediate treatment are the most effective methods for combating pancreatic cancer. Secreted proteins involved in cell-cell interactions can alter tumor microenvironments, which can protect and promote tumor progression by rendering therapeutics ineffective^3^. In light of this, these secreted proteins can be utilized as biomarkers to identify cancer and its disease stage in the clinic.

Our previous studies have showed that the extracted dissociation factor (DF) from the culture medium (10% fetal bovine serum, FBS) of highly invasive hamster pancreatic cancer cells (PC-1.0) could induce invasion/metastasis in weak invasive hamster pancreatic cancer cells (PC-1). Our results suggest that DF may play an important role in pancreatic cancer metastasis, particularly at an early disease stage^4^,^5^,^6^,^7^. However, due to the interference of FBS, candidate DF proteins involved in pancreatic cell invasion/metastasis remain unknown. Recently, the metabolic labelling, protein equalization, protein fractionation, and filter-aided sample preparation (FASP) strategy (MLEFF) has been developed and successfully applied in the comprehensive and quantitative secretome analysis.

In this work, we identified a total of 161 significantly changed secreted proteins from the conditioned medium of two pancreatic cancer cell lines. By integrated analysis of our data and publicly available bioinformatics resources, we found that a combination panel consisting of CDH3, PLAU, and LFNG might improve the prognosis of overall pancreatic cancer survival. In addition, many of the regulated proteins identified in our study play key roles in tumor metastasis, but the role of extracellular proteins may be more directly function for tumor metastasis. The results from this study shed light on pancreatic cancer invasion and metastasis, and may provide insight into the development of novel therapeutic strategies.

## Results

### Identification and quantification of cell culture secretome

**Figure 1:**
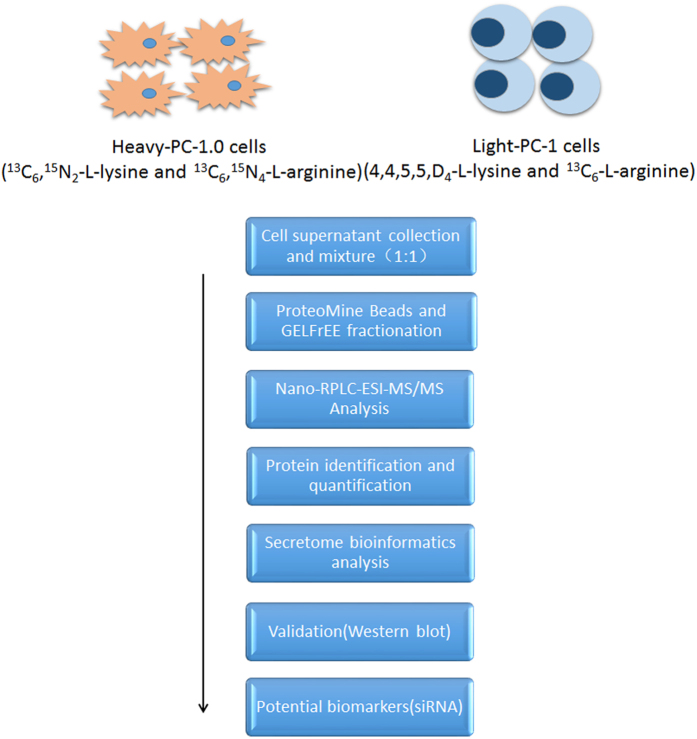
Schematic of study protocol. Conditioned media were collected, proteins were precipitated, and secretome proteins were identified using LC–MS/MS.

To characterize secreted proteins associated with pancreatic cancer metastasis/invasion, we compared the conditioned media (CM) of two homologous cell lines (PC-1.0 vs. PC-1) with different metastasis potential. In this study, CM were analyzed using SILAC-based quantitative proteomics. CMs from PC-1.0 (heavy labeled) and PC-1 (medium labeled) were harvested individually, mixed at equal volumes, and three independent replicates were analyzed (Fig. 1). The mass spectra*.raw files were searched against both the Chinese hamster and human proteome sequence databases, and overlapping proteins were identified. Following proteomic analysis, we identified 761 and 672 cell-originated proteins (CoPros, SILAC-labelled) in the Chinese hamster and human proteome sequence databases, respectively. Of them, 314 and 267 proteins were quantified in all three replicates and searched against both databases. Among them, We observed 13 proteins up-regulated and 148 proteins down-regulated in PC-1.0 CM compared to PC-1 CM. Table 1 lists top 10 of all proteins with a >1.5 fold-change in expression between cell lines (Supplemental Table 1 lists all identified proteins from the SILAC experiment and Supplemental Table 2 lists 161 differentially expressed proteins (DEPs) with >1.5 fold change in expression).

### Gene ontology and cellular location of secreted proteins in pancreatic cancer

**Figure 2:**
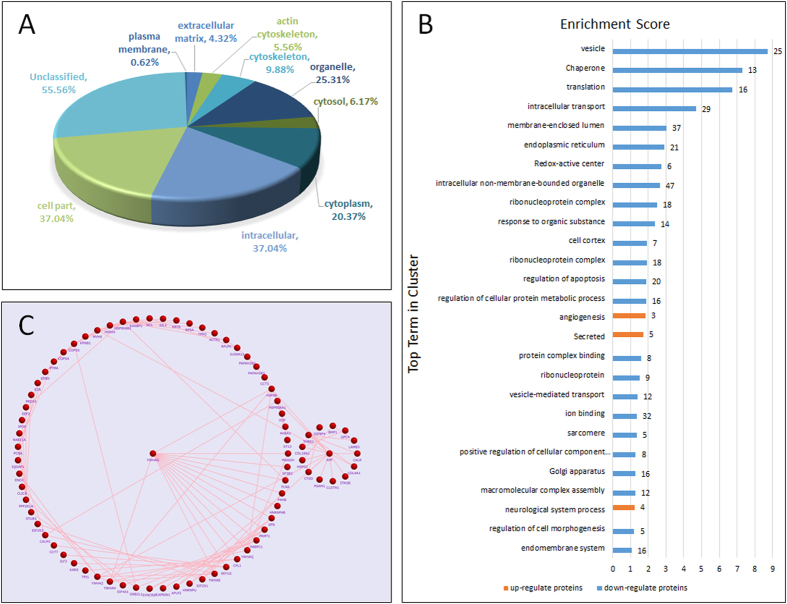
Analysis of the location, biological function, and protein-protein interactions of quantified proteins. (**A**) Analysis of the location of differentially secreted proteins was based on GO. (**B**) Biological function analysis of the 161 differentially expressed proteins. (**C**) Protein-protein interaction analysis of the 161 differentially expressed proteins enriched by Funrich.

We analyzed the subcellular localization of DEPs. The results from the GO analysis were as follows (% of total proteins, number of proteins, P-value, fold-enrichment): extracellular proteins (4.32%, 7, 3.87E-02, 4.9), actin cytoskeleton (5.56%, 9, 7.10E-03, 4.85), cytoskeleton (9.88%, 16, 1.86E-03, 3.32), organelle (25.31%, 41, 5.68E-04, 2), cytosol (6.17%, 10, 1.57E-02, 3.95), cytoplasm (20.37%, 33, 3.33E-03, 2.05), intracellular (37.04%, 60, 5.11E-06, 1.93), cell part (37.04%, 60, 1.19E-05, 1.89), plasma membrane (0.62%, 1, 1.80E-02, 0.2) and Unclassified (55.56%, 90, 0.00E+00, 0.79) (Fig. 2A). To determine if these proteins were secreted, data from the current study was searched against web-based bioinformatic tools (SignalP 4.1 and SecretomeP 2.0). Remarkably, 47 out of the total 161 proteins contained signal peptides, and 35 showed a non-classical protein secretion pathway without signal peptide (Supplemental Table S3). Thus, a total of 82 proteins were considered secreted proteins via different pathways. For data mining comparisons, we used our list of identified proteins to compare against the Exocarta database. Using Exocarta we found that 153 proteins matched our protein list, suggesting that some of these proteins may be secreted via extracellular vesicles. Only SEPT11, SNU13, HS3ST1, and MAGOH were undetected using this database.

### Functional and signaling pathway analysis, and validation of secreted proteins

**Figure 3:**
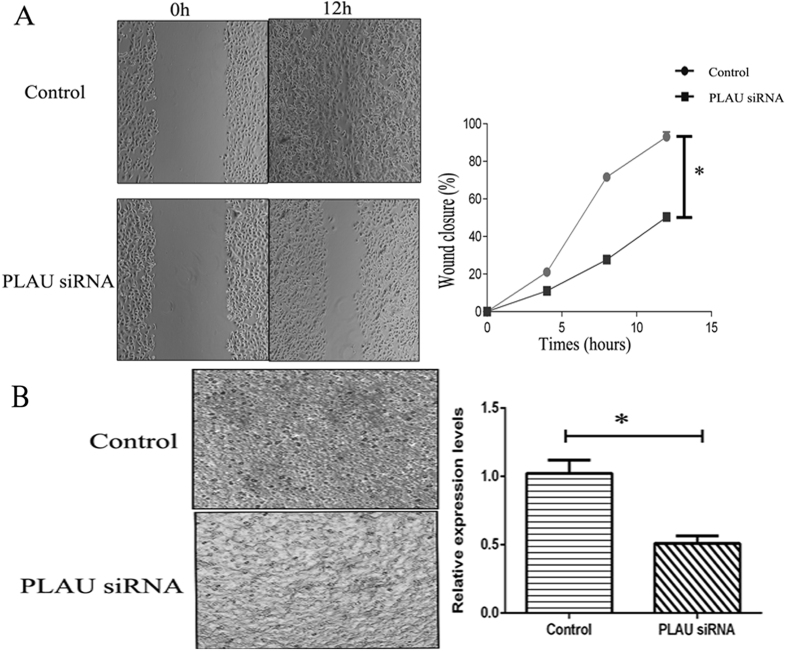
PLAU play a major role in cell migration and invasion of PC-1.0 Metastatic. (**A**) Effect of PLAU on the migration after using siRNA in highly metastatic PC-1.0 cells. The cells were incubated for 24 h. The percentage migration was calculated and graphed. *Compared with the PC-1.0, *P*-value < 0.01. (n = 3) (**B**) Quantification of transwell assay for treated group and control group. Cells were counted in triplicate wells and in three identical experiments, *Compared with the PC-1.0, *P*-value < 0.01. (n = 3).

To identify altered biological characteristics that may play a role in pancreatic cancer metastasis, DEPs were analyzed using DAVID software. According to the results from the analysis of enriched GO clusters, it was apparent that up-regulated proteins in biological processes are secreted and primarily involved in angiogenesis. As for down-regulated proteins, the majority of them were involved in vesicle, chaperone, translation, and intracellular transport (Fig. 2B, Supplemental Table S4). There was a cluster composed of 13 proteins related with cell motion (ACTR2, ARPC2, TPM3, NRP2, YWHAE, CAPZB, and ARPC1B, etc.). We further analyzed the interactions between proteins and relevant signaling pathways using Funrich software. The top five networks were related to insulin-mediated glucose transport, p38 signaling mediated by Mitogen-activated protein kinases-activated protein (MAPKAP) kinases, proteoglycan syndecan-mediated signaling events, glypican pathway, and protein metabolism (Table 2 and Supplemental Table S5). As shown in Fig. 2C, YWHAG and APP participate in cell apoptosis and adhesion, respectively, but have a central role in protein-protein interactions. Of the networks, the majority are related to proteoglycan, which plays an important role in establishing the tumor microenvironment. Using the Perseus program, we identified 6 DEPs including CDH3, DnaJ homolog subfamily B member 11 (DNAJB11), PLAU, palmitoyl-protein thioesterase 1 (PPT1), cathepsin D (CTSD), and β-1,3-*N*-acetylglucosaminyltransferase lunatic fringe (LFNG). All of these proteins participate in cell communication and protein metabolism. To understand functional relationships of differential alteration in highly metastatic PC-1.0 cells, we have selected one protein (PLAU) and performed the corresponding invasion and migration assays. A strong inhibition of migration was observed for PLAU (Fig. 3A), and regarding invasion, PLAU also caused a significant reduction in the invasion ability (Fig. 3B). Collectively, these findings indicate that DEPs are related to cell motility, cell-cell signaling, and growth, all of which are necessary for cancer growth and invasion.

**Figure 4:**
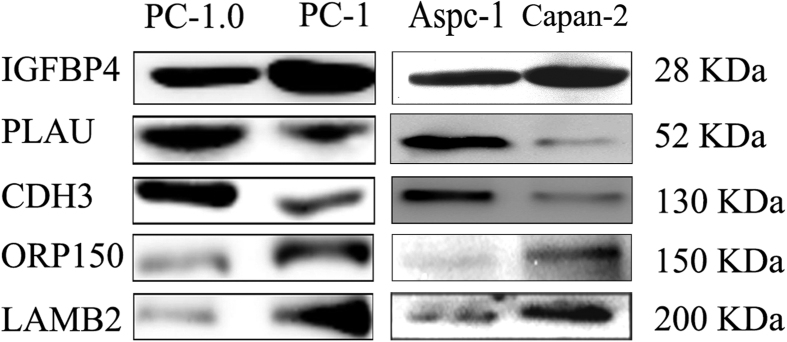
Western blots reveal differential expression of the secretomes of highly (PC-1.0, Aspc-1) and weakly (PC-1, Capan-2) invasive and metastatic pancreatic cancer cells. As shown, the results were consistent with the MS quantification data.

Five differentially expressed candidates (two up-regulated and three down-regulated) with available antibodies were selected for initial validation using western blot analyses of concentrated supernatants (Fig. 4). Western blot results were consistent with the MS quantification data.

### The combined strategy discovered pancreatic cancer biomarker

**Figure 5:**
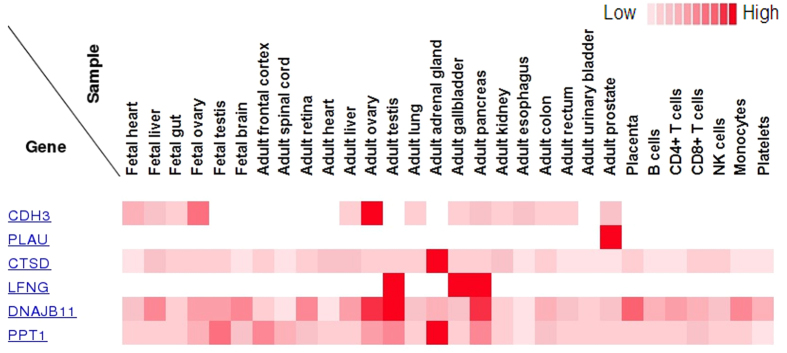
Protein expression of six genes observed in normal pancreatic tissue searched against the human proteome map database.

**Figure 6:**
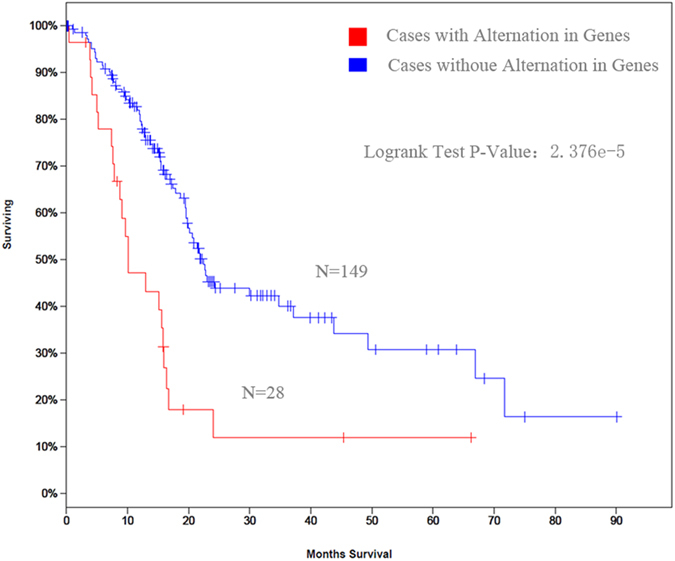
Overall survival of pancreatic cancer asosicated with CDH3, LFNG, and PLAU mRNA expression. Using cBioCancer Genomics Portal database, a panel consisting of these three genes had worse overall survival than other combinations (log-rank test *p*-value: 2.376 × 10^−5^).

One of the goals of this study was to discover the potential pancreatic cancer biomarkers by evaluating proteins that were up-regulated in the PC-1.0 metastatic cell line. In this study, we identified 4 of 6 DEPs that have previously been associated with pancreatic cancer (CDH3, PLAU, CTSD, and LFNG)^8^,^9^,^10^,^11^. To the best of our knowledge, DNAJB11 and PPT1 have not been previously reported to play a role in pancreatic cancer. DNAJB11 localizes in the endoplasmic reticulum and is involved in protein folding^12^. It was up-regulated 16.20-fold in the secretome of PC-1.0 cells. PPT1 is a small glycoprotein associated with infantile neuronal ceroid lipofuscinosis (INCL). Cho S *et al*. have reported that the overexpression of PPT1 protects against apoptosis in neuroblastoma cells^13^. We observed that PPT1 was up-regulated by 2.07-fold in the pancreatic cancer secretome. Future studies will be necessary to elucidate the roles of DNAJB11 and PPT1 in pancreatic tumorigenesis. Furthermore, we investigated six proteins from published proteomic data. As shown in Fig. 5 and Table 3, the expression of the six proteins associated with pancreatic cancer was different in the human protein atlas database and the human proteome map database. So more samples should be given to verify the real expression. In addition, these studies were performed based on intracellular proteins. From an extracellular perspective, these secreted proteins may play more roles in tumor microenvironment and metastasis than the corresponding intracellular protein. Furthermore, using the Human Plasma Peptide Atlas database, we found that 98 proteins had been previously expressed in human plasma and may be candidate biomarkers for pancreatic cancer prevention, diagnosis, and prognosis (Supplemental Table S6). Lastly, we searched each candidate protein against the cBio Cancer Genomics Portal database, which predicts overall survival using publicly available genomic data. Our search was set to a total of 186 pancreatic adenocarcinoma samples, and mRNA expression with a z-score threshold ±2.0. As a result, combinations of CDH3, LFNG, and PLAU were significantly associated with overall survival (log-rank test *p*-value: 2.376 × 10^−5^; Fig. 6) compared to other protein combinations and cancers (e.g. hepatocellular carcinoma, colorectal adenocarcinoma, and lung adenocarcinoma; Supplemental Figure). Our results indicated that CTSD, DNAJB11, and PPT1 were not related with overall survival of pancreatic cancer (data not shown). Hence, our data suggests that a protein biomarker panel consisting of three proteins may significantly improve prognosis of pancreatic cancer.

## Discussion

Tumor microenvironment is important for the development and metastasis of cancer cells^14^,^15^. Among the multitude of factors that affect the tumor microenvironment, the secretome has recently received more attention. When cancer cells secrete proteins into the extracellular environment, some may play a role in facilitating tumor formation. Previous studies have showed that secretome analysis may be an effective approach for identification of cancer biomarkers, which is critical for improving diagnosis and monitoring treatment^16^,^17^. The majority of the current secretome analysis utilize serum-free culture to avoid the interference of FBS. However, serum-starvation is clearly proved has great potential to interfere the experimental results and affect subsequent conclusions, although important discoveries have been revealed using this strategy^18^. In this work, the secretome profiling of two pancreatic cancer cells (PC-1 and PC-1.0) were performed in the presence of serum using a MLEFF approach.

In our previous study, a secreted protein produced by PC-1.0 cells, named DF, was identified, which affects the microenvironment and growth morphology of homologous PC-1 cell lines. In addition, DF is different from other scatter factors that have been described previously^6^, and appears to be a unique scatter factor. Therefore, understanding the molecular changes that underlie the differentially expressed secretome of two homologous pancreatic cancer cell lines is of critical importance. Over the past few years, we have investigated the alterations of two pancreatic cancer cell lines at the genomic and phosphoproteomic levels^9^,^19^. Through integration strategy, we observed that some proteins are expressed at the same level in both the genome and secretome (e.g. MMP13). Alternatively, we observed that some of the findings from the genomic analysis are in contrast to the corresponding secretome analysis (e.g. PLAU). These results suggest that secreted proteins may experience a different splice, which is in low abundance and too difficult to detect. Furthermore, similar to DF, PLAU may induce the dissociation of the PC-1 cell, since we observed no difference in PLAU expression between the invasive front and center of the pancreatic cancer tissue^20^. It will be important to further study the role of DF in pancreatic cancer the future.

We further analyzed the pancreatic cancer secretome by combining publicly available sources. Among six of the proteins we identified, the mRNA expression of three of these proteins was associated with overall survival. In addition, the majority of proteins identified in our study were considered secreted proteins via the exosome pathway. Stated differently, these proteins could be detected in pancreatic cancer patient plasma. A previously published study by Chan A *et al*. reported that biomarker panels could greatly improve sensitivity and specificity for early diagnosis of PDAC^21^. To assess the usefulness of this panel of proteins (CDH3, LFNG, and PLAU) in pancreatic cancer diagnostics, sera from pancreatic cancer patients and their kindred should be analyzed. The study will be more clinically value for personalized medicine.

This study has some limitations. First, a few proteins were identified multiple times with our approach, however, these proteins were not quantitative data. Among them, we evaluated the expression of LAMB2. Our analysis showed that the expression of LAMB2 was different among our two pancreatic cancer cell lines. Thus, to create a completely exhaustive list of candidate biomarkers, some proteins, such as LAMB2, may need to be further analyzed. Second, the overall survival of pancreatic cancer was based on mRNA expression. However, with respect to clinical diagnosis, the protein expression would be more useful (e.g. prostate specific antigen, PSA). Thus, the overall survival of pancreatic cancer based on protein expression levels should be investigated in the future. Finally, the differences between species of cell lines should not be ignored, and the correlation of secretome alterations and clinically features should be further validated.

In conclusion, the secretome of pancreatic cancer cell lines under physiological conditions was achieved. Moreover, the identified proteins may play an important role in pancreatic cancer metastasis and invasion, ultimately being utilized as biomarkers for therapuetic intervention.

## Materials and Methods

### Reagents and Materials

Trypsin (bovine pancreas), formic acid, trifluoroacetic acid, urea, protease inhibitor cocktail, dithiothreitol, trichloroacetic acid, acetone, and iodoacetamide were purchased from Sigma–Aldrich (St. Louis, MO, USA). Acetonitrile was ordered from Merck & Co. (Kenilworth, NJ, USA). Deionized water was purified by a Milli-Q system (Millipore, MA, USA) and all other chemicals utilized were analytical-grade.

We purchased bicinchoninic acid (BCA) protein assay kit from Beyotime Institute of Biotechnology (Tianjin, China), ProteoMiner protein enrichment kits from Bio-Rad (Hercules, CA, USA), and syringe and ultracentrifugal filters (3 kDa and10 kDa molecular weight cut-off (MWCO), respectively) from Millipore (MA, USA). Rabbit polyclonal antibodies raised against human epitopes cadherin 3, type 1, P-cadherin (placental) (CDH3; Santa Cruz Biotechnology, TX, USA), laminin, beta 2 (laminin S) (LAMB2; Santa Cruz Biotechnology, TX, USA), oxygen-regulated protein 150 kDa (Abcam, Cambridge, MA, USA), insulin-like growth factor binding protein 4 (R&D Systems, MN, USA), plasminogen activator, urokinase (PLAU; R&D Systems) were used as primary antibodies. Horseradish peroxidase-conjugated secondary antibodies or FITC-labeled fluorescent antibodies (Santa Cruz Biotechnology, TX, USA) were used as secondary antibodies for western blotting. PLAU siRNA were purchased from Santa Cruz Biotechnology (Dallas, TX, USA).

### Cell lines and cell culture

In this study, we evaluated two hamster pancreatic cancer cell lines with different invasion/metastatic potential. *In vitro*, PC-1.0 cells are mainly single cells, whereas PC-1 cells grow in an island-like formation. *In vivo*, local invasion by PC-1.0 cells and local expansion of PC-1 cells were observed^22^. Human pancreatic cancer cell lines Aspc-1 and Capan-2, which have morphological and functional characteristics similar to PC-1.0 and PC-1 cells, respectively, were used to determine if the results from hamster cells coincide with human pancreatic cancer cell lines.

These cells were incubated in Roswell Park Memorial Institute medium (RPMI-1640; Gibco-BRL, NY, USA) supplemented with 10% fetal bovine serum (FBS) (Bioserum, Canterbury, Victoria, Australia), 100 U/mL penicillin G, and 100 μg/mL streptomycin at 37 °C in a humidified atmosphere of 5% CO_2_,95% air. For the stable isotope labeling by amino acids in cell culture (SILAC) experiment, we labeled PC-1.0 cells with heavy [^13^C_6_, ^15^N_2_] L-lysine and [^13^C_6_, ^15^N_4_] L-arginine, and PC-1 cells with light [4, 4, 5, 5-D_4_] L-lysine and [^13^C_6_] L-arginine (Thermo Fisher Scientific, MA, USA) in RPMI-1640 medium. Cells were supplemented with 10% dialyzed FBS (Thermo Fisher Scientific) and cultured for eight passages to achieve maximum labeling.

### Protein Extraction

Pancreatic cancer cells were cultured in RPMI-1640 medium containing 10% FBS. Following eight passages, with cells at nearly 80% confluence, media was replaced with fresh media, and cells were incubated for an additional 24 h. Supernatants were collected for secretome analysis. To extract proteins, we followed a previously published method with minor modifications^23^. Briefly, dead cells and debris were removed by centrifuging the sample at 500 × *g* and 3000 × *g* for 15 min at 4 °C, respectively. Protease inhibitor was added at an equal volume to the supernatant, and samples were filtrated using a syringe filter. Filtrated samples were added to a 3 kDa ultrafiltration tube (15 mL; Millipore) and centrifuged at 3500 × *g* in 4 °C, for 120 min. Following centrifugation, ProteoMiner protein enrichment kits were used according to the manufacturer’s protocol to enrich low-abundance proteins. The resulting solution was concentrated using a 3 kDa ultrafiltration tube, and washed three times with 8 M urea. Proteins were divided into 10 fractions using a GELFrEE 8100 Fractionation System (Expedeon, San Diego, CA, USA). A BCA protein assay was used to measure protein concentrations. Lastly, proteins were processed using the FASP method^24^, and peptides were manually collected and analyzed with a 1D nano-rapid reverse-phase liquid chromatography (RPLC)-tandem mass spectrometry (MS/MS) on a Q-Exactive MS (Thermo Fisher Scientific) coupled with an Ultimate 3000 (Dionex, Sunnyvale, CA, USA) nano-liquid chromatography (LC) system. All digests were desalted, lyophilized, and stored at −80 °C prior to analysis.

### Nano-RPLC-MS/MS Analysis

A nano-flow liquid chromatograph (Ultimate 3000; Dionex) with a Q-Exactive MS (Thermo Fisher Scientific) was used to analyze protein fractions. Mobile phases consisted of phases A (98% H_2_O and 2% acetonitrile with 0.1% formic acid) and B (2% H_2_O and 98% acetonitrile with 0.1% formic acid). Peptides were loaded onto a C18 trap column (150 μm i.d. × 5 cm), and then separated by a capillary separation column (75 μm i.d. × 15 cm) at a flow rate of 120 μL/min. All columns were prepared in-house for LC-MS/MS analysis. The gradient of the mobile phase B was set as follows: 10 min of 0–6% B, 100 min of 6–35% B, 10 min of 35–80% B, and 10 min maintained at 80% B. The temperature of the ion transfer capillary was set at 275 °C with a spray voltage of 2.5 kV. The Q-Exactive, performed at a resolution of 70,000 (MS1) and 17,500 (MS2), was performed in the positive ion data-dependent mode using one MS scan followed by 15 MS/MS scans with a 20 seconds exclusion window and a normalized collision energy of 28% for fragmentation. The automatic gain control (AGC) was set to 1 × 10^6^ and 1 × 10^5^ for MS/MS. Total ion chromatograms and mass spectra were recorded from at a range of 300–1800 *m*/*z* with Xcalibur software (ver. 2.1.0; Thermo Fisher Scientific). Samples were analyzed in triplicate.

### Database Searching

Raw data files generated from LC–MS/MS analyses were converted to Mascot generic format (mgf) using Proteome Discoverer (PD, ver. 1.4.1.14; Thermo Fisher Scientific). Proteome Discover used workflow from the Mascot search engine (ver. 2.3.2) and searched against the UniProtKB *Cricetulus griseus* (Chinese hamster; release 2015_06; 34,957 entries) and Homo sapiens (human; release 2015_04; 42,121 entries) proteome sequence databases. These databases were utilized instead of the UniProtKB *Mesocricetus auratus* (Golden hamster) database, because it only contained 942 sequences, which may have introduced bias. Mass tolerances for Q-Exactive were set at 7 p.p.m. for parent ions and 20 p.p.m. for fragments. Carbamidomethylation (+57.0215 Da) served as the fixed modification, and oxidation of methionine (+15.999 Da) and protein N-terminal acetylations (+42.0106 Da) were variable modifications. Light (Lys4, +4.0251 Da and Arg6, +6.0201 Da) and heavy labels (Lys8, +8.0142 Da and Arg10, +10.0083 Da) were also considered variable modifications. The other search parameters were set as follows: enzyme, trypsin; 2 missing cleavages sites. The false discovery rate (FDR) was set as less than 1%. For protein quantification, default parameters were used.

### Bioinformatic Analysis

From three replicates, all proteins were reliably identified and quantified based on *P*-values < 0.05. Protein ratios with a 1.5-fold change were considered to be differentially expressed proteins (DEPs). To assemble the compendium, we mapped differentially expressed hamster proteins to their human homologs in the human UniProt database on the basis of protein names. For subcellular localization, DEPs were identified using PANTHER for GO analysis (http://pantherdb.org/)^7^. We performed a biological functions analysis of the DEPs using DAVID (http://david.abcc.ncifcrf.gov/)^25^, and Funrich software^26^. Meanwhile, SignalP 4.1 (http://www.cbs.dtu.dk/services/SignalP/, probability >0.90)^27^, SecretomeP 2.0 (NN-score >0.50)^28^, and the Exocarta database (http://exocarta.org/)^29^ were used to rapidly predict if the quantified proteins were secretory proteins. *In silico* secretome prediction may aid in mining the data, although shortcomings exist, accuracy may be verified using a different assay. In addition, to ascertain whether identified proteins were previously detected in plasma, we searched the Human Plasma Peptide Atlas (1% FDR; contains 1929 proteins; www.peptideatlas.org/hupo/hppp/)^30^. The cBio Cancer Genomics Portal was used to determine the relationship between candidate proteins and pancreatic cancer using publicly available genomic data on PDAC^31^. Associations between normal pancreatic tissue and cancerous tissue at the gene and protein expression levels for candidate proteins were obtained from the human proteome map^32^ and the human protein atlas database^33^.

### Western Blot Analysis

Western blot analysis was performed to evaluate secreted proteins. Cultures were centrifuged for 2 min at 10,000 × *g* and supernatants were filtered through a 0.22 μm syringe (Millipore). Protease inhibitor was added and the conditioned medium was concentrated by centrifugation in Amicon Ultracel-3K units, flash frozen, and stored at −80 °C until further analysis. Secreted proteins were dissolved in 4 μL of 5 × sodium dodecyl sulfate polyacrylamide gel electrophoresis (SDS-PAGE) buffer, boiled for 3 min, and separated by SDS-PAGE. Protein concentrations were determined using a BCA assay and proteins were identified by western blotting as previously described^34^.

### *In vitro* invasion and migration assays

PC-1.0 cells were transiently transfected with PLAU siRNA using Lipofectamine 2000 (Invitrogen, Grand Island, NY). Invasion Transwell assays and wound healing migration assay were performed as described previously^19^.

### Statistics Analysis

Statistical analysis was performed using Perseus version 1.5.1.6^35^. Overall survival and progression-free survival were estimated using the Kaplan–Meier method and compared using a log-rank test. If not stated otherwise, comparisons among groups were analyzed using a two-tailed Student’s *t*-test. *P*-values ≤ 0.05 were considered to be statistically significant.

## Additional Information

**How to cite this article**: Liu, P. *et al*. Quantitative secretomic analysis of pancreatic cancer cells in serum-containing conditioned medium. *Sci. Rep.* **6**, 37606; doi: 10.1038/srep37606 (2016).

**Publisher’s note**: Springer Nature remains neutral with regard to jurisdictional claims in published maps and institutional affiliations.

## Supplementary Material

Supplemental Info

Supplemental Table S1

Supplemental Table S2

Supplemental Table S3

Supplemental Table S4

Supplemental Table S5

Supplemental Table S6

Supplemental Figure

## Figures and Tables

**Table 1:** Proteins with a ≥1.5-fold difference in expression between the PC-1.0 (heavy label) and PC-1 (light label) cell lines.(TOP10).

gene name	Exp.1		Exp.2		Exp.3	
	H/L count	H/L ratio	H/L count	H/L ratio	H/L count	H/L ratio
CDH3	5	30.149	8	43.839	7	38.917
DNAJB11	2	9.704	1	11.225	3	39.007
PLAU	2	7.45	3	3.597	1	14.742
APLP2	91	5.275	92	4.962	80	5.358
MMP13	2	18.339	1	0.088	1	75.037
LAMB3	52	3.861	47	3.808	51	3.504
COL18A1	46	2.864	46	2.619	42	3.111
SEMA3B	4	2.651	5	2.183	5	2.204
B4GALT4	12	2.339	12	2.526	12	2.096
NCL	10	2.044	15	2.245	16	2.094

**Table 2:** Interactions of differentially expressed proteins enriched by Funrich.

pathway	Gene Count	P-value	Gene
p38 signaling mediated by MAPKAP kinases	6	4.09E-07	YWHAQ; SFN; YWHAG etc
Proteoglycan syndecan-mediated signaling events	41	4.50E-07	HSP90AA1; SFN; THBS1 etc
Insulin-mediated glucose transport	7	1.15E-07	YWHAZ; YWHAH; YWHAQ etc
Glypican pathway	39	3.49E-06	YWHAZ; COPS5; YWHAH etc
Metabolism of proteins	15	4.42E-06	RPSA; CALR; RPLP0 etc
Beta1 integrin cell surface interactions	39	4.49E-06	THBS1; NCL; KPNB1 etc
VEGF and VEGFR signaling network	38	5.14E-06	ACTR3; MMP13; KRT8 etc
Translation	10	6.46E-06	RPSA; RPLP0; PABPC1 etc
TNF alpha/NF-kB	12	6.90E-06	PSMD7; HSP90AA1; YWHAG etc
Signaling mediated by p38-alpha and p38-beta	7	7.43E-06	YWHAG; KRT8; YWHAE etc

**Table 3:** The expression of six proteins referred by the human protein atlas database.

Gene	RNA expression in normal tissue(FPKM)	RNA expression in cancer cell line(FPKM)	Protein staining in normal tissue	Protein staining in cancer tissue
CDH3	0	18	high	medium
PLAU	2	483	medium	low
CTSD	74	1769	medium	medium
LFNG	10	19	medium	low
DNAJB11	34	145	medium	high
PPT1	7	91	high	high
