# Supplementary material for: Quantitative secretomic analysis of pancreatic cancer cells in serum-containing conditioned medium
Source: Sci Rep. 2016 Nov 21;6:37606. doi: 10.1038/srep37606 (PMC5116583; doi:10.1038/srep37606)
Supplement: Supplemental Info [file srep37606-s1.doc]

**Quantitative secretomic analysis of pancreatic cancer cells in serum-containing conditioned medium**

Peng Liu^1^, Yejing Weng^2^, Zhigang Sui^2^, Yunhao Wu^1^, Xiangli Meng^1^, Mengwei Wu^1^, Haoyi Jin^1^, Xiaodong Tan^1,*^, Lihua Zhang^2,*^, Yukui Zhang^2^

The first two authors are co-first author.

^1^ 1st Department of general surgery, Shengjing Hospital, China Medical University, Shenyang 110004, China

^2^ Key Lab of Separation Sciences for Analytical Chemistry, National Chromatographic R. & A. Center, Dalian Institute of Chemical Physics, Chinese Academy of Sciences, Dalian 116023, China

^*^ Corresponding Author: Xiaodong Tan, 1st Department of general surgey, Shengjing Hospital, China Medical University, Shenyang 110004, China. Phone: +86-24-9661531111, E-mail:tanxdcmu@163.com; Lihua Zhang, Dalian Institute of Chemical Physics, Chinese Academy of Science, Dalian 116023, China. Fax: 86-411-84379720, E-mail: LihuaZhang@dicp.ac.cn

**Supplementary information**

Supplemental Table S1: A complete list of all the proteins identified in this study.

Supplemental Table S2: List of 161 proteins differentially expression by PC-1.0 and PC-1 pancreatic cancer cells with fold change > 1.5.

Supplemental Table S3. In silico analysis with Exocarta database of the 161 differentially expression proteins to correlate non-classical secretion with their presence in extracellular vesicles.

Supplemental Table S4. GO term analysis of the 161 differentially expression proteins by DAVID.

Supplemental Table S5. The interaction of differentially expression proteins were enriched by Funrich.

Supplemental Table S6. The 161 differentially expression proteins were searched against Human plasm database.

Supplemental Figure: Overall survival of CDH3, LFNG and PLAU at mRNA level in other cancer was displayed.
