# Supplementary material for: Quantitative secretomic analysis of pancreatic cancer cells in serum-containing conditioned medium
Source: Sci Rep. 2016 Nov 21;6:37606. doi: 10.1038/srep37606 (PMC5116583; doi:10.1038/srep37606)
Supplement: Supplemental Figure [file srep37606-s8.docx]

**Quantitative secretomic analysis of pancreatic cancer cells in serum-containing conditioned medium**

Peng Liu^1^, Yejing Weng^2^, Zhigang Sui^2^, Yunhao Wu^1^, Xiangli Meng^1^, Mengwei Wu^1^, Haoyi Jin^1^, Xiaodong Tan^1,*^, Lihua Zhang^2,*^, Yukui Zhang^2^

The first two authors are co-first author.

^1^ 1st Department of general surgery, Shengjing Hospital, China Medical University, Shenyang 110004, China

^2^ Key Lab of Separation Sciences for Analytical Chemistry, National Chromatographic R. & A. Center, Dalian Institute of Chemical Physics, Chinese Academy of Sciences, Dalian 116023, China


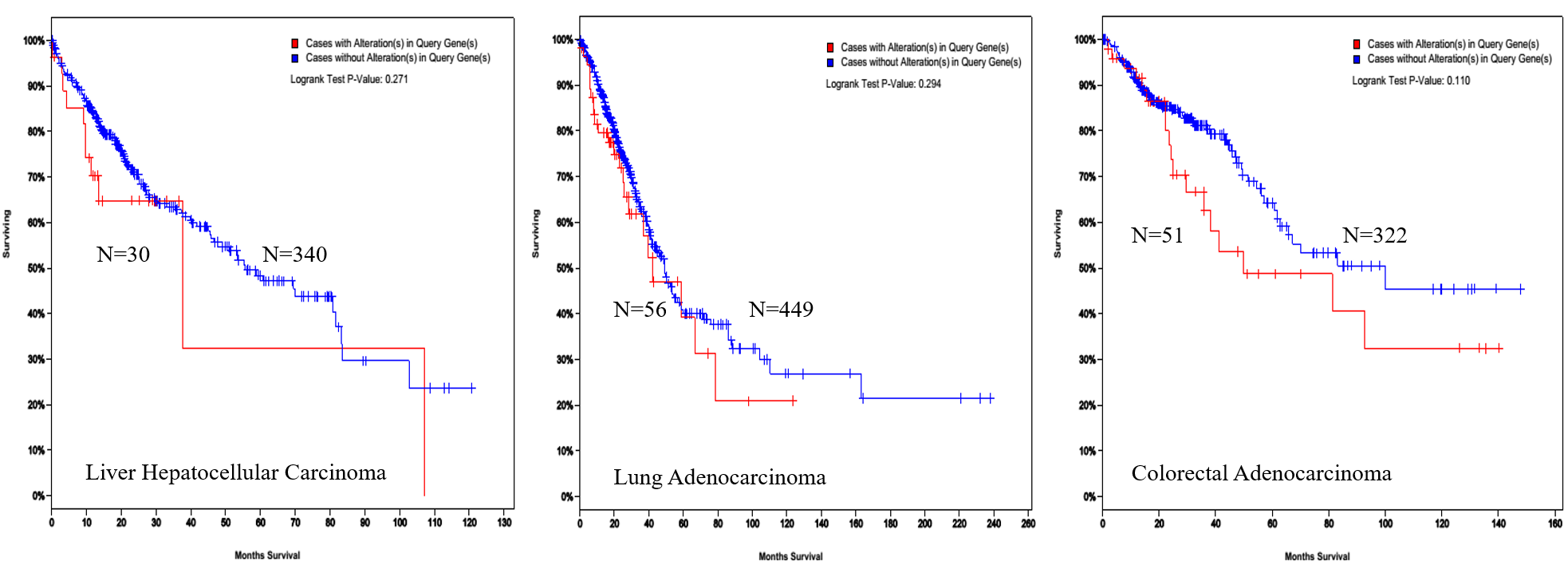
^*^ Corresponding Author: Xiaodong Tan, 1st Department of general surgey, Shengjing Hospital, China Medical University, Shenyang 110004, China. Phone: +86-24-9661531111, E-mail:tanxdcmu@163.com; Lihua Zhang, Dalian Institute of Chemical Physics, Chinese Academy of Science, Dalian 116023, China. Fax: 86-411-84379720, E-mail: LihuaZhang@dicp.ac.cn
